# The utility of cMet as a diagnostic tissue biomarker in primary colorectal cancer

**DOI:** 10.1111/iep.12395

**Published:** 2021-05-05

**Authors:** Gemma R. Armstrong, Mohammed Ibrahim Khot, Jim P. Tiernan, Nick P. West, Sarah L. Perry, Tom I. Maisey, Thomas A. Hughes, David G. Jayne

**Affiliations:** ^1^ Leeds Institute of Medical Research St. Jamess University Hospital University of Leeds Leeds UK; ^2^ Leeds Teaching Hospitals NHS Trust Leeds UK

**Keywords:** biomarker, cMet, colorectal cancer, immunohistochemistry

## Abstract

The transmembrane protein, cMet, is thought to be overexpressed and activated in colorectal cancer (CRC). This study explored its potential as a diagnostic tissue biomarker for CRC in a large human CRC tissue collection obtained from a randomized clinical trial.

Tissue microarrays of matched normal colorectal epithelium and primary cancer were prepared from specimens obtained from 280 patients recruited to the MRC CLASICC trial (ISRCTN 74883561) and interrogated using immunohistochemistry for cMet expression. The distribution and intensity of immunopositivity was graded using a validated, semiquantifiable score, and differences in median scores analysed using the Wilcoxon signedrank test. A receiver operating characteristic (ROC) curve was plotted to measure the diagnostic accuracy of cMet as a biomarker in CRC.

Epithelial cell membrane expression of cMet differed significantly between CRC and normal colorectal tissue: median 12.00 (Interquartile range (IQR) 615) versus median 6.00 (IQR 2.7012.00) respectively (*P*=<.0001). ROCAUC analysis of cMet expression yielded a CRC diagnostic probability of 0.66 (95% CI: 0.61 to 0.70; *P*<.0001). A score of 14.50 showed high specificity at 85.32% (95% CI 80.33%89.45%) but sensitivity of only 30.92% (CI 25.37%36.90%).

Thus cMet is consistently overexpressed in human CRC as compared to normal colorectal epithelium tissue. cMet expression may have a role in diagnosis and prognostication if combined with other biomarkers.

## 1 INTRODUCTION

Colorectal cancer (CRC) is the fourth most common cancer in the UK, with around 42,000 newly diagnosed cases each year.^1^ The majority of CRC is associated with mutations in oncogenes, tumour suppressor genes and DNA mismatch repair genes. These mutations initiate and propagate the adenomacarcinoma sequence towards the development of malignant disease in the majority of cases.^2^ Patient survival is strongly influenced by tumour stage at the time of initial diagnosis, with 1year overall survival rates of 98% vs. 40% for American Joint Committee on Cancer stage 1 and stage 4 respectively.^3^

Biomarkers are routinely used in clinical practice for diagnosis, to guide prognostication and to predict response to treatment. Expression of the oncofoetal antigen, carcinoembryonic antigen (CEA), is assessed as a blood serum biomarker of CRC and is routinely used to guide postoperative surveillance for recurrent disease.^4^, ^5^ The absence of activating Kirsten rat sarcoma viral oncogene (*KRAS*) and neuroblastoma RAS viral oncogene homolog (*NRAS*) mutations is confirmed prior to commencing antiEGFR (antiepidermal growth factor receptor) monoclonal antibody in advanced CRC ^6^, ^7^, ^8^, ^9^ due to the negative predictive effect of these mutation; however, most wildtype patients still show no significant response to these agents. High expression of the ligands of EGFR, epiregulin (EREG) and amphiregulin (AREG) at the RNA level was deemed highly predictive of therapeutic benefit with the antiEGFR agent panitumumab in KRAS/NRAS wildtype metastatic CRC in the PICCOLO trial (International Standard Randomised Controlled Trial Number (ISRCTN): 93248876)^7^, ^10^; however, routine assessment of these or other predictive tissue biomarkers is not routinely performed in CRC.

The receptor tyrosine kinase, mesenchymalepithelial transition factor (cMet), is a transmembrane protein and key mediator of many cellular processes. The expression of cMet receptor is primarily localized to epithelial tissues and is activated by the ligand hepatocyte growth factor (HGF). Under normal conditions, the formation of this receptorligand complex is vital for many processes, including embryogenesis, tissue regeneration, disruption of celltocell adhesion, promotion of morphogenesis and differentiation.^11^, ^12^, ^13^ The expression of cMet has been found to be upregulated in many malignancies, including nonsmallcell lung cancer,^14^ hepatocellular carcinoma^15^ and CRC.^16^ Increased expression of cMet protein in cancer can be stimulated by physiological hypoxia. This adaptive response gives malignant cells a survival benefit within hypoxic tumour microenvironments.^17^ In recent years, there has been increasing interest in cMet as a diagnostic and prognostic biomarker, with several studies suggesting that over expression is linked to poorer survival in CRC.^18^

Our study is the first of its kind to investigate the potential for cMet to serve as a diagnostic biomarker for CRC by evaluating its expression in a tissue obtained from a randomized controlled trial of resected CRC.

## 2 MATERIALS AND METHODS

### 2.1 Trial cohort and ethical approval

The Medical Research Council (MRC) CLASICC trial (ISRCTN 74883561)^19^, ^20^ recruited patients with potentially curable CRC to either laparoscopic or open resection. Consent was obtained from participating patients for residual samples of normal colorectal epithelium and cancer to be stored for future research. Ethical approval for use of tissue from the MRC CLASICC trial was obtained from the National Research Ethics Service (LondonDulwich Committee), reference 12/LO/1327 in 2012. Formalin fixed paraffin embedded (FFPE) tissue samples from a representative subgroup of 280 patients were incorporated into seven anonymized tissue microarrays (TMA). Three tumour cores and three matched normal mucosa cores (each 0.6mm diameter) from the most representative area of the archived resection block were incorporated into the TMA.^5^ Control tissue samples of archival placenta, ovary, prostate, kidney, epididymis, liver, gallbladder, stomach, oesophagus, pancreas, small bowel, lung and muscle were incorporated into the TMA margins for comparative and orientation purposes.

### 2.2 Immunohistochemical detection of cmet protein in tissue samples

Sections were prepared from the TMA blocks onto SuperFrost Plus Adhesion glass slides (Thermo Fisher Scientific, Altrincham, UK) at 5m thickness. These were deparaffinized in xylene and rehydrated in decreasing concentration gradients of ethanol. Heatinduced antigen retrieval was performed by placing the slides into citric acid buffer solution, adjusted to pH 6.2, and microwaved for 10minutes at 900W. Slides were then incubated with an anticMet monoclonal antibody (1:250, Recombinant anticMet antibody [EP1454Y], Abcam PLC, Cambridge, UK) for one hour at room temperature. Following incubation with the primary anticMet antibody, slides were incubated with SignalStain Boost IHC Detection Reagent (HRP, Rabbit; Cell Signalling Technology Inc, Leiden, Netherlands) for 30minutes in a humidified chamber. The SignalStain 3,3'diaminobenzidine (DAB) Substrate Kit (Cell Signalling Technology Inc, Leiden, The Netherlands) was used, and the chromogen solution applied for 10minutes for antibody visualization. In between each incubation step, the slides were washed in a dilute solution of trisbuffered saline solution. Slides were counterstained with Mayer's Haematoxylin solution (SigmaAldrich Scientific Inc, Gillingham, UK), dehydrated and mounted on glass cover slips.

### 2.3 Imaging

Whole slide images were produced at x40 magnification with the Leica BioSystems Aperio AT2 whole slide scanner (Leica Microsystems Ltd, Milton Keynes, UK). Slide images were viewed at x40 magnification and assessed using Aperio ImageScope v12.4.0.5043 (Leica BioSystems Pathology Imaging Ltd, Milton Keynes, UK).

### 2.4 Scoring cMet expression

The distribution and intensity of cMet protein expression was assessed by a trained research fellow, using a previously validated scale.^5^, ^21^, ^22^, ^23^ cMet protein immunopositivity in epithelial cell membranes from each tissue core was scored. The intensity of the chromogenic reaction was quantified with a numerical scale between 0 and 3 (0=no detectable expression, 1=mild expression, 2=moderate expression and 3=strong expression). The distribution of membranous cMet immunopositivity was determined by calculating the percentage of tumour or normal epithelial cells deemed positive as a percentage of all of the tumour/normal epithelial cells in the core total area. For tumour cores, noncancerous tissue was excluded from the analysis. (The distribution was graded 0 = <5%, 1=5%20%, 2=21%40%, 3=41%60%, 4=61%80%, 5=81%100%.) The intensity and distribution scores generated were multiplied to provide a compound cMet expression score. Cores with insufficient tumour or normal epithelial tissue for assessment were not included. Control TMA slides were prepared to exclude fixation dependent observations. In total, 263 tumour and 253 normal epithelium core triplicates (or pairs if a single core not assessable) were suitable for analysis and 242 matched tumournormal tissue paired samples (86.43% of pairs) were included in the final analysis.

The scoring criteria were appraised and approved by all authors in a consensus meeting prior to initiating the scoring process. Representative examples of cMet expression at each distribution score 0 to 5 and intensity score 0 to 3 were approved by the group and available for reference throughout the scoring process. Independent verification was provided by a second research fellow with appropriate experience of IHC scoring beforehand. Ten per cent of the 242 matched tumournormal paired samples were selected at random and presented for secondary verification. Where discrepancy arose between the reviewers a consensus decision was reached.

### 2.5 Statistical analysis

Microsoft Excel and Graphpad Prism 7 (GraphPad V7.04 Software, Inc, California, USA) were used for all data handling and statistical analyses. Wilcoxon matchedpairs signedrank test was used to compare the median rank difference in scores between the matched (tumour and normal tissue) TMA samples. The interquartile range (IQR) and twotailed *P* value are reported. A statistical significance threshold *of P=*<.05 was applied. The Wilcoxon matchedpairs test estimate of the 95% confidence interval (95% C.I) of the median difference bound by the IQR were calculated. This method used binominal probabilities to predict the 95% C.I around the median and is therefore asymmetrical around the median value provided.^24^ To account for uncertainty in the data caused by missing core sample pairs, a sensitivity analysis assuming the highest cMet expression (compound score 15) for all missing data fields was performed.

A receiver operating characteristic curve (ROC curve) was plotted to determine the diagnostic accuracy of cMet protein expression in CRC. The sensitivity of cMet for the detection of CRC (true positive rate) was plotted as a function of the specificity (true negative rate) and used to calculate varying decision cutoff thresholds. The area under the curve (AUC) was calculated to numerically quantify the diagnostic accuracy of various cMet expression scores in discriminating normal epithelial from cancerous tissue.

## 3 RESULTS

### 3.1 Population demographics of the CLASICC trial participants

The key demographic and clinicopathological characteristics of the 280 patient cohort from the CLASICC trial are shown inTable 1.^5^, ^19^, ^20^ Entire sample triplicates of seventeen tumour (6.07%) and 27 (9.64%) normal epithelial tissue cores were ungradable.

**TABLE 1:** Key clinical and pathological features of CLASICC TMA patient cohort. Adapted from.^5^, ^23^, ^24^ Staging performed according to TNM version 5

Characteristic	Number of cases (%)N*=280*
Age range: 3393years Mean age: 68.9years	
Male	144 (51.4)
Female	136 (48.6)
pT stage	
1	10 (3.6)
2	54 (19.3)
3	161 (57.5)
4	52 (18.6)
Unknown	3 (1.1)

Note

The utility of cMet as a diagnostic tissue biomarker in primary colorectal cancer.

### 3.2 cMet expression

The normality test was applied and confirmed the populations were not sampled from a normal distribution, and a nonparametric test was required: skewness 0.61 and 0.29 and kurtosis 1.1 and 1.3 for tumour and normal tissue respectively (*P*<.0001).

**FIGURE 1:**
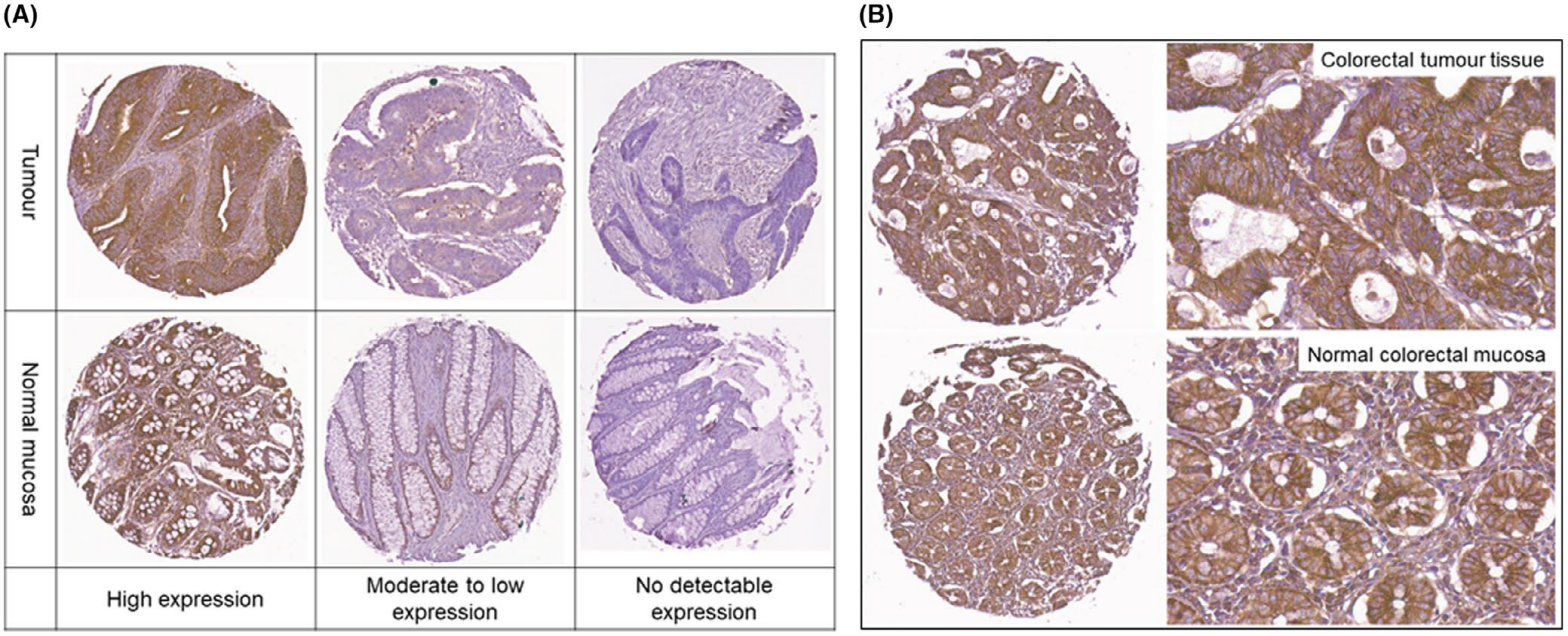
A, example of cMet expression in matched participant normal epithelium and colorectal cancer tissue TMA at each scoring threshold. Whole core imaged at x13 magnification for illustrative purposes. B, Example of high cMet expression in normal and colorectal cancer tissue at x13 and x40 magnification

Variable cMet immunopositivity was observed across the tissue core population. CRC demonstrated more frequent strong cMet expression as compared to normal epithelial mucosa. High expression was defined as an overall cMet expression intensity score of 15 and was found in 30.80% (81/263) of CRC samples as compared to 12.25% (31/253) in normal tissue samples (*P*<.0001). No detectable cMet expression with a composite cMet expression score of 0 was found in 7.90% (20/253) of normal colorectal tissue samples versus 3.42% (9/263) in matched tumours (*P*<.0001). Representative examples of cMet expression are shown in Figure1.

The median tissue cMet IHC expression scores were 12.0 for tumour (IQR 6.0 to 15.0) and 6.0 for normal epithelium (IQR 2.7 to 12.0) *P* =<0.0001 (Table2). Wilcoxon matchedpairs test showed a significant difference between the median expression scores of malignant and normal colorectal tissue, median difference 2.33 (IQR 0.25 to 7.7) and with a difference of sum of signedrank W score of 15397 (*P*<.0001) (n=242 pairs).

**TABLE 2:** Summary of statistical analyses of TMA cMet expression by IHC score (tumour versus normal tissue)

	Tumour	Normal	Difference
Number of pairs	242		
Minimum	0.00	0.00	14.00
25% Percentile	6.00	2.80	0.25
Median	12.00	6.00	2.33
75% Percentile	15.00	12.00	7.70
Maximum	15.00	15.00	15.00

Wilcoxon Matchedpairs SignedRank Test			
*P* value (two tailed)	<.0001	<.0001	
95% C.I of median difference			1.00 to 3.50
IQR median difference			0.33 to 7.70
Sum of signed ranks (W)		15397	
Sum of positive rank		22274	
Sum of negative rank		6877	

### 3.3 Accuracy of cMet as a colorectal cancer diagnostic biomarker

**FIGURE 2:**
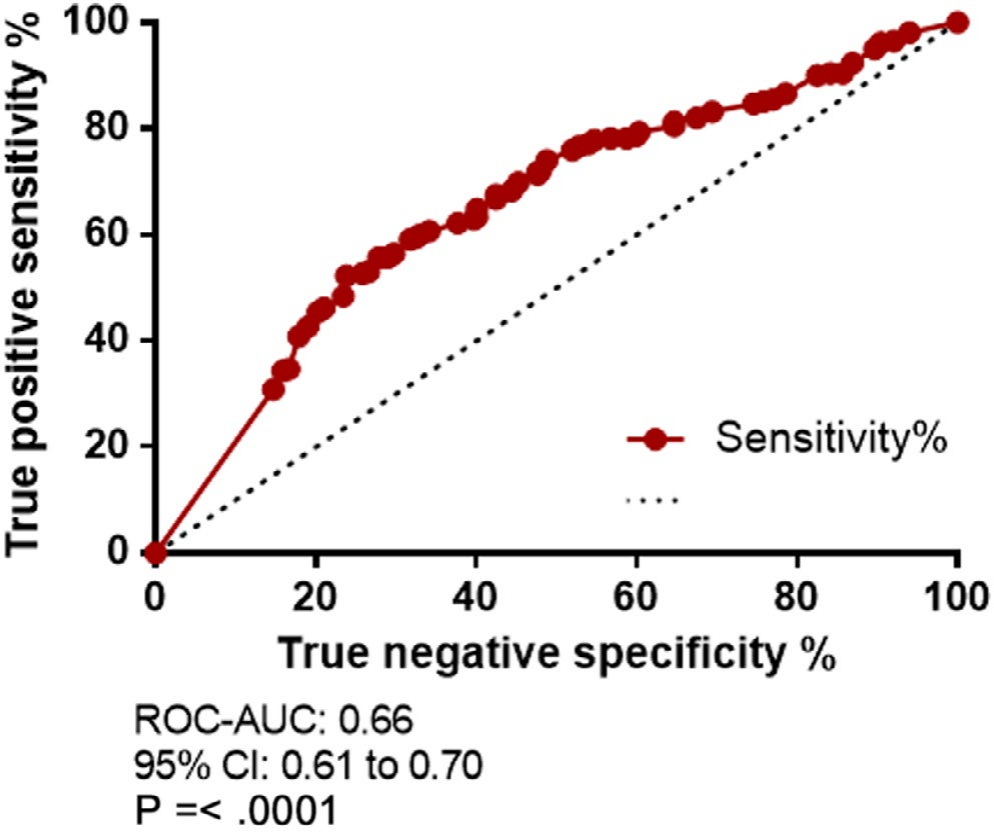
Receiver operating characteristics (ROC) curve and area under curve (AUC) for cMet expression. Sensitivity plotted as a function of specificity as percentage at various decision thresholds for cMet as a diagnostic biomarker in colorectal cancer detection

The receiver operating characteristics (ROC) curve and the area under curve (AUC) of the sensitivity and specificity of cMet expression at various thresholds are shown in Figure2. The ROCAUC score yielded an overall diagnostic accuracy predictive probability score of 0.66 (95% CI: 0.61 to 0.70; *P*=<.0001).

A total expression score of14.50 yielded a high specificity of 85.32% (C.I 80.33% to 89.45%); however, there was a significant tradeoff with sensitivity of 30.92% (C.I 25.37% to 36.90%) and a likelihood ratio of 2.11. A combined score of the total sensitivity and specificity from each cutoff generated by the ROC curve was calculated. An overall cMet expression score cutoff of11.58, yielded the greatest combined true positive and true negative rate with a sensitivity of 55.73% (95% CI 49.48% to 61.84%) and specificity of 71.83% (95% CI 65.84% to 77.29%), likelihood ratio 1.98. The highest likelihood ratio of 2.29 was observed with a cMet expression score of13.17: sensitivity 40.84% (95% CI 34.83% to 47.06%) and specificity 82.14% (95% CI 76.85% to 86.67%).

### 3.4 Sensitivity analysis

The sensitivity analysis assumed the highest level of cMet expression for all missing data (compound score 15). This confirmed a significant difference in median score between the matched tissue samples, 12.30 for tumour (IQR 7.5815.00; *P*=<.0001) and 10.00 for normal epithelium (IQR 6.0013.30; *P*=<.0001). Similarly, the ROCAUC analysis showed an AUC value of 0.60 (95% CI 0.55 to 0.64; *P*=<.0001). However, intense cMet immunopositivity (expression score 14.50) yielded a slightly lower CRC predictive value specificity of 78.47% (95% CI 73.12% to 83.19%) and sensitivity of 33.94% (95% CI 28.35% to 39.88%) with a likelihood ratio 1.19.

## 4 DISCUSSION

Oncogenic activation of the transmembrane protein, cMet, has been reported to induce multiple intracellular changes leading to the development and progression of cancers.^23^, ^25^, ^26^, ^27^ Overexpression of cMet has been reported in several gastrointestinal malignancies, including CRC.^24^ Previous small studies have identified the overexpression of cMet in metastatic CRC as a predictor of poor overall and progressionfree survival.^23^, ^26^, ^27^ We quantified the expression of cMet protein in matched tumour and normal epithelial tissue samples from participants of the MRC CLASICC trial. Our study is unique in its utilization of tissue from a multicentre RCT study cohort to examine a novel biomarker, both in its size and representation of the CRC patient population in the UK.

We adopted a previously validated scoring system^5^, ^21^, ^22^, ^23^ in this study. Tumour samples had a significantly increased median cMet protein expression, as compared to matched normal mucosa. The cMet protein plays a vital homeostatic role in epithelial cell function; therefore, the rate of high cmet expression (12.25%) by normal mucosa cores (compared to 30.80% in CRC cores) was entirely expected and observed in previous immunohistochemical studies of cMet expression.^28^ Our data support the results of smaller studies; Takeuchi et al ^29^ confirmed cMet mRNA copies were significantly elevated in a small sample of CRC patients relative to normal colonic epithelium. Interestingly, their study of 36 patients also identified mRNA copy levels correlated with primary CRC depth of invasion and lymph node status.

Examination of the diagnostic accuracy of cMet expression in CRC tissue samples confirmed that a total cMet expression score of 12 or over, yielded the greatest combined specificity and sensitivity for the detection of CRC, at 76.19% and 52.29% respectively. The differential expression and diagnostic accuracy ROC curve assessment indicates that cMet may have a role in the diagnosis of CRC and could be a possible therapeutic target.

Previous studies have confirmed the potential of tissue CEA as a diagnostic and surveillance biomarker and the CEA receptor protein as an imaging target in CRC.^5^, ^27^, ^30^ *Tiernan et al* ^5^ used a similar scoring method to this study and identified increased CEA expression in CRC tissue, when compared to corresponding normal tissue. Although we found that cMet lacked the diagnostic accuracy of CEA, it outperformed the other biomarkers explored in the Tiernan study: epidermal growth factor receptor (EGFR), Folate receptor alpha (FR) and tumourassociated glycoprotein 72 (TAG72). Formation of the protooncogene cMetHGF ligand complex activates several downstream signalling cascades and induces nuclear transcription and cellular transformation. This can include activation of the *RAS* protein family.^9^, ^11^, ^23^ *KRAS* and *NRAS* mutation status is assessed prior to treatment with antiEGFR agents in CRC. It is possible cMet interacts with other known oncogenic pathways in the development of, and chemotherapy resistance seen in CRC *Yoon et al* confirmed strong correlation between cMet and a related downstream nonreceptor tyrosine kinase, focal adhesion kinase (FAK) expression Hscores in CRC TMA. With the related macrophagestimulating protein receptor (MST1R), a threeprotein risk stratification model of CRC confirmed high FAK, low cMET and low MST1R protein levels showed the worst progressionfree survival and a high risk of early progression of disease. In combination, cMet may be beneficial in the diagnosis or prognostication of CRC, especially in combination with other novel biomarkers.

## 5 CONCLUSION

Immunohistochemical analysis of cMet expression in human tissue samples showed a significant differential expression between matched normal colorectal epithelium and CRC. Malignant tissue consistently overexpressed cMet protein, relative to normal tissue. Strong cMet expression only yielded a true negative rate of 30.92%. With such a high false positive rate, the diagnostic and prognostic utility of cMet in CRC may be limited. Further evaluation of cMet in conjunction with other biomarkers is required and may have a prognostic, surveillance or response to treatment predictive role.

## CONFLICT OF INTEREST

None declared.

## AUTHORS' CONTRIBUTIONS

G R Armstrong designed and conducted the experiment, analysed the results and drafted the manuscript with guidance from N P West and J P Tiernan and G R Armstrong. J Tiernan previously designed and validated a similar IHC grading classification system and assisted with adapting the method for this project. M I Khot, S L Perry and T I Maisey provided training on laboratory techniques, and T A Hughes, N P West and J P Tiernan assisted G R Armstrong with interpretation of the results. All authors saw and approved the final version of the manuscript.

## ETHICS APPROVAL AND CONSENT TO PARTICIPATE

Tissue was obtained from participants of the MRC CLASICC randomized controlled trial (Medical Research Council Conventional versus LaparoscopicAssisted Surgery in Colorectal Cancer) (Trial identifiersClinicalTrials.gov number NCT00003354 and Clinical Trial Number ISRCTN 74883561). Approval process described previously.^24^ Ethical approval for tissue research was obtained from the National Research Ethics Service (LondonDulwich Committee), reference 12/LO/1327.

## CONSENT FOR PUBLICATION

The authors obtained at the time of CLASICC trial enrolment by local investigators and under National Research Ethics Service (LondonDulwich Committee), reference 12/LO/1327 approval for tissue research.

## Data Availability

The datasets analysed and results presented in this study are available from the corresponding author on reasonable request.
